# AI-generated Feedback Following Social Robotic Virtual Patient Interactions and Medical Student Performance: Nonrandomized Quasi-Experimental Study

**DOI:** 10.2196/90368

**Published:** 2026-03-25

**Authors:** Alexander Borg, Jonathan Schiött, William Ivegren, Cidem Gentline, Viking Huss, Anna Margareta Hugelius, Benjamin Jobs, Mini Ruiz, Samuel Edelbring, Carina Georg, Gabriel Skantze, Ioannis Parodis

**Affiliations:** 1 Division of Rheumatology Department of Medicine Solna Karolinska Institutet, Karolinska University Hospital, and Center for Molecular Medicine (CMM) Solna Sweden; 2 Division of Clinical Epidemiology Department of Medicine Solna Karolinska Institutet and Karolinska University Hospital Solna Sweden; 3 Department of Clinical Science, Intervention and Technology Karolinska Institutet Stockholm, Stockholm Sweden; 4 Department of Educational Sciences and Arts Faculty of Philosophy Mälardalen University Västerås, Västmanland Sweden; 5 School of Health Sciences Örebro University Örebro, Örebro Sweden; 6 Department of Neurobiology, Care Sciences and Society Karolinska Institutet Stockholm, Stockholm Sweden; 7 Division of Speech Music and Hearing Royal Institute of Technology (KTH) Stockholm, Stockholm Sweden; 8 Department of Rheumatology Faculty of Medicine and Health Örebro University Örebro, Örebro Sweden

**Keywords:** virtual patients, medical education, clinical reasoning, large language models, social robotics, artificial intelligence, AI, educational technology

## Abstract

**Background:**

Virtual patients (VPs) demonstrate effectiveness in improving clinical reasoning skills; however, traditional VP platforms often lack individualized feedback mechanisms. Advances in large language models (LLMs) enable automated analysis of student-VP interactions, providing scalable feedback on clinical performance. While artificial intelligence (AI)–enhanced social robotic VP platforms show promise for clinical reasoning training, no studies have examined whether AI-generated feedback integrated in such platforms improves clinical performance in standardized assessments.

**Objective:**

This study evaluated whether AI-generated postconsultation feedback integrated into social robotic VP interactions improves medical students’ clinical performance, emphasizing medical history taking and communication.

**Methods:**

A quasi-experimental study with 115 sixth-semester medical students (N=157, 73.2% of eligible students) was conducted at Karolinska Institutet, Stockholm, Sweden, during spring 2025. Students were allocated by hospital site to receive (n=61, 53%) or not receive (n=54, 46.9%) AI-generated feedback following interactions with a Social AI-Enhanced Robotic Interface. All students completed 9 VP cases; the intervention group received approximately 1 page of structured feedback after each VP case. The feedback system used multiple LLMs following a 2-stage algorithm: assessing student-VP dialogues using an assessment rubric, then generating structured feedback on history-taking performance. Both groups participated in case-specific follow-up seminars led by consultant rheumatologists following each VP encounter. Clinical performance was assessed through an 8-minute objective structured clinical examination (OSCE)-based evaluation, with a standardized patient portraying axial spondylarthritis, evaluated by a blinded consultant rheumatologist using a 10-point rubric across 5 domains: communication at consultation start, generic medical history, targeted medical history, diagnostics and management reasoning, and communication at consultation end.

**Results:**

Students receiving AI-generated feedback achieved significantly higher total OSCE scores (mean 7.39, SD 0.86 vs mean 6.68, SD 1.04 points; mean difference 0.70; 95% CI 0.35-1.06; *P*<.001; Cohen *d*=0.74). Domain-specific analysis revealed significant improvement in generic medical history after Bonferroni correction (mean 2.46, SD 0.65 vs mean 2.03, SD 0.79 points; *P*=.004; *r*=0.27), while other domains showed no significant differences: communication at start (*P*=.13; *r*=0.14), targeted medical history taking (*P*=.60; *r*=0.05), diagnostics and management (*P*=.14; *r*=0.14), and communication at consultation end (*P*=.31; *r*=0.09). Pass rates were significantly higher in the feedback group (96.7% vs 79.6%; odds ratio 7.55, 95% CI 1.51-72.2; *P*=.006), with a number needed to assess of 6 students, that is, for every 6 students receiving feedback, 1 additional student passed the assessment.

**Conclusions:**

AI-generated feedback following social robotic VP interactions significantly improved medical students’ OSCE-based performance, particularly in generic medical history taking. These findings support integrating validated AI feedback systems as a supplement to expert-led teaching during VP simulations for clinical training and demonstrate the feasibility of scalable, automated feedback in medical education. The domain-specific improvements in generic medical history highlight the importance of targeted, competency-specific feedback design in VP platforms.

## Introduction

Clinical reasoning (CR) represents a fundamental cognitive process that guides diagnostic and therapeutic decisions in clinical practice [1,2]. Virtual patients (VPs) have emerged as valuable educational tools for developing CR skills in medical education, providing safe environments for practicing patient interactions [3,4]. While VPs demonstrate effectiveness in improving CR skills, traditional VP platforms often lack sophisticated and individualized feedback mechanisms, potentially limiting skill transfer to real-world clinical encounters [5,6]. The integration of artificial intelligence (AI) within VP simulations addresses this limitation, with recent systematic reviews demonstrating positive effects on CR skills, particularly in case-specific domains such as data gathering and diagnostic accuracy [6,7].

Social robots are physically embodied, autonomous systems designed to interact with humans following social behaviors and communication norms [8]. Social robotics represents a promising frontier in simulation-based interactions, with educational applications showing enhanced engagement and learning outcomes across various disciplines [9]. However, no studies have examined the integration of AI-generated feedback within social robotic VPs or evaluated whether such feedback improves objectively measured clinical performance.

Diagnostic errors represent a significant challenge in health care, with inadequate history taking identified as a leading contributor, as medical history alone can often aid in establishing a correct diagnosis [10,11]. Scalable approaches to improving the quality of medical history–taking training therefore carry implications beyond educational outcomes, extending to actual patient benefit [12]. Feedback represents a cornerstone of effective medical education, particularly in developing CR skills [13,14]. Effective feedback should be specific, timely, and actionable, addressing both the content and process of CR [15]. Recent advances in large language models (LLMs) enable automated analysis of student-VP interactions, providing scalable feedback on medical history taking and diagnostic reasoning [16,17]. Studies using AI-generated feedback have demonstrated improved diagnostic accuracy and enhanced systematism during clinical problem solving [18].

We have developed a Social AI-Enhanced Robotic Interface (SARI) for VP encounters and demonstrated that this interface provides superior design characteristics for CR training compared with conventional computer-based VP platforms [19-22]. While technological advances such as AI-enhanced VPs show promise, ensuring the transfer of skills from simulated to real clinical encounters remains a critical challenge in VP education [4]. Objective structured clinical examinations (OSCEs) enable standardized assessment of application of skills in controlled settings and provide a structured bridge between simulated training environments and real-world clinical performance [23-25]. In our prior work, we examined the added value of AI-enhanced social robotic VPs compared with conventional computer-based VP simulations, focusing on medical students’ self-reported perceptions [19-22]. However, these studies did not include objective performance measures, and the VP platforms lacked systematic feedback on students’ CR processes.

While AI-enhanced social robotic VPs show promise for CR training, no studies have examined whether integrated AI-generated feedback translates into improved clinical performance as measured by means of standardized assessments. This study aimed to evaluate whether the integration of AI-generated postconsultation feedback within social robotic VP training enhances medical students’ performance in CR skills with emphasis on history taking and communication, assessed through an OSCE-based evaluation with a standardized patient. We used a quasi-experimental design to test our hypothesis that students receiving AI-generated feedback following VP encounters would achieve higher OSCE scores compared with students completing the same VP training without such feedback. The findings are primarily relevant for medical educators, curriculum designers, and researchers developing AI-enhanced simulation-based training platforms.

## Methods

### Overview

We conducted a quasi-experimental study to evaluate whether AI-generated feedback following social robotic VP interaction improves medical students’ clinical performance in medical history taking. The study was conducted at Karolinska Institutet, Stockholm, Sweden, during spring 2025, following the Transparent Reporting of Evaluations with Nonrandomized Designs reporting guidelines [26].

The study participants (N=115) completed an established virtual outpatient clinic educational activity within rheumatology, which involves interaction with 9 VP cases using the SARI [19-22] and the computer-based virtual interactive case system [27] for CR training. Students were allocated either to the intervention group (n=61, 53%), where AI-generated feedback was provided after each VP encounter with the SARI, or to the control group (n=54, 46.9%), where students completed identical cases without receiving AI-generated feedback. During a total of 6 weeks, students from 4 different hospital sites attended their week-long rheumatology clinical rotation, with each student based at Karolinska University Hospital Solna for the virtual outpatient clinic. The virtual outpatient clinic spanned one and a half days in total during this week. There was no crossover, and each student was exposed to either the intervention or control condition. Allocation was determined by hospital site to facilitate scheduling, which rotated weekly between intervention and control conditions to avoid potential selection bias owing to hospital site specifics. A flowchart of study participants and allocation is illustrated in Figure S1 in Multimedia Appendix 1.

Immediately following completion of all 9 VP cases at the end of the virtual outpatient clinic, students were evaluated for their performance in CR through an OSCE-based assessment, with emphasis placed on medical history taking and communication. The primary outcome was a comparison of total OSCE scores between the 2 groups, with secondary outcomes including domain-specific OSCE performance across 5 subdomains and pass or fail rates using a predetermined threshold. The OSCE-based rubric is presented in Figure S2 in Multimedia Appendix 1.

### Study Participants

All sixth-semester medical students attending the virtual outpatient clinic during their rheumatology clinical rotation were eligible and invited to take part in the study. Of 157 eligible students, 115 (73.2%) were included in the study. Participation was voluntary, with students providing informed consent before enrollment. There were no formal inclusion criteria, instead all students in the sixth semester were asked to participate. Students were excluded if they were unable to attend the scheduled OSCE-like assessment or did not provide informed consent.

The virtual outpatient clinic includes interaction with 9 VP cases, followed by case-specific seminars with consultant rheumatologists. Students work in pairs or small groups of three, with one student taking the lead in each patient interaction, consistent with evidence supporting collaborative VP learning for CR training [28]. Before this educational activity, students receive instructions and training in structured medical history taking, covering both generic aspects and case-specific aspects with relevance for the discipline of rheumatology (Figure S3 in Multimedia Appendix 1). Students were based at 4 clinical sites in the Stockholm region, that is, Karolinska University Hospital Solna, Karolinska University Hospital Huddinge, Danderyd Hospital, and Södersjukhuset, but the virtual outpatient clinic took place at Karolinska University Hospital Solna for all students.

### AI-Generated Feedback

We developed and validated an AI-powered feedback system integrated with the SARI. The system uses multiple LLMs from OpenAI to analyze transcribed student-VP dialogues and generate structured feedback on medical history–taking performance.

The feedback algorithm followed a 2-stage design, developed using historical SARI interaction data from previous semesters, and validated through consultant rheumatologist review. The evaluation framework covers communication skills; generic and rheumatology-specific aspects of medical history taking, including systematic symptom enquiry, pain characterization, and functional assessment; and selected domains of CR essential for diagnostic and management purposes.

The first stage of the feedback algorithm is an assessment model that evaluates student-VP dialogues using a predefined rubric developed in collaboration with consultant rheumatologists. The model was iteratively refined before the study and has been described previously [29]. Initial algorithm outputs were compared against expert assessments across 30 student-VP interaction dialogues that were graded twice by each rheumatologist. Final validation demonstrated a high degree of agreement between AI-generated assessment and assessment provided by consultant rheumatologists (81.8% accuracy, Cohen k=0.68) [29].

The second stage of the feedback algorithm involves generating a feedback output based on the stage-1 assessment. Students received approximately 1 page of structured written feedback immediately after completing each VP encounter with the SARI (but not the virtual interactive case system), across a total of 5 VP scenarios. This feedback focused on medical history taking within the context of rheumatology and included constructive comments with examples covering general history taking, specific symptom enquiries, and systematic assessment of the VPs. No feedback was provided on diagnostic accuracy or the appropriateness of proposed management steps. The 2-stage feedback algorithm, the stage 2 prompt used to generate feedback from the stage 1 assessment, and an example of AI-generated feedback are illustrated in Figures S4-S6 in Multimedia Appendix 1.

### The Social AI-Enhanced Robotic Interface

The SARI is a VP modality concept developed and implemented by our research group and combines AI using LLMs with a social robot [19-22]. In this specific context, the SARI combines an LLM from OpenAI (GPT-4o-mini) [30] with a social robot from Furhat Robotics, which encompasses a back-projected face on a robotic head and is capable of producing humanlike facial expressions, natural head movements, and lip-synchronized speech [31]. The principles of VP case development in the SARI have been described previously and include a prompt that combines a patient description with the last 10 dialogue turns to avoid repetition (Figure S7 in Multimedia Appendix 1). Improvements to the SARI are made between study periods following feedback from students participating in the rheumatology clinical placements and with the release of updated versions of LLMs. Refinements implemented before the investigation for this study included the use of a newer LLM (GPT 4o-mini), which also enabled removal of the previously required LED dialogue indicator. These improvements were important based on a previous qualitative study wherein students perceived the dialogue with the previous version of SARI somewhat mechanical at times [20]. The SARI configuration was frozen before data collection commenced and remained unchanged throughout the study.

VP cases are initiated with a patient introducing themselves to a medical student, describing why they are there. Afterward, the dialogue is free, and students explore the virtual environment until they feel that they can proceed to preliminary diagnostics and the suggestion of a management plan. Students engaged with the SARI through natural speech dialogue, with all interactions being audio-recorded and transcribed automatically within the system for the generation of AI-powered feedback. The transcribed dialogue was available to all students following interaction with the SARI. Along with the cases, students receive a standard laboratory test panel, including case-specific laboratory test results along with the corresponding reference values. All VP cases were in English to also accommodate visiting international exchange students who participate in clinical rotations in rheumatology at Karolinska University Hospital within the frame of separate educational activities or courses. However, all sixth-semester students who participated in this study were Swedish speaking, with most having Swedish as their mother tongue.

### Data Collection

Immediately after completing the virtual outpatient clinic, participating students underwent an OSCE-like evaluation designed to assess performance in medical history taking and CR. The 8-minute evaluation involved interaction with a standardized patient, portrayed by 2 engineering coauthors of this study without medical training. They were instructed to portray a patient with axial spondylarthritis and received written case information in advance. Performance was assessed independently by 1 of 2 consultants in rheumatology using a standardized rubric, with the assessors being blinded to whether students had received AI-generated feedback as a part of the intervention or were allocated to the control group. Unlike the VP interactions during the virtual outpatient clinic, this OSCE-like evaluation was conducted in Swedish. Instructions for the assessment, provided to students and standardized patients, are presented in Figures S8 and S9 in Multimedia Appendix 1.

The assessment used a 10-point scale across 5 domains with differential weighting reflecting their relative importance during a clinical encounter: communication at consultation start (0-3 points), generic medical history (0-3.5 points), targeted medical history (0-1.5 points), diagnostics and management reasoning (0-1 point), and communication at consultation end (0-1 point). The rubric and domain weighting was developed and determined collaboratively with consultant rheumatologists based on established Entrustable Professional Activity frameworks [32], reflecting the emphasis on history-taking competencies in the virtual outpatient clinic. The rubric underwent iterative refinement through pilot testing with consultant rheumatologists who reviewed and provided feedback on the scoring criteria, domain definitions, and descriptions to ensure clarity and clinical relevance. A pass threshold of 6 out of 10 points was predetermined based on clinical competency standards and local Entrustable Professional Activity framework requirements (Figure S2 in Multimedia Appendix 1).

### Statistical Analysis

Sample size was determined by the number of available students during clinical rotations in rheumatology, representing a convenience sample of all available participants. Baseline characteristics were compared between groups using 2-tailed independent samples *t* tests for continuous variables and Fisher exact tests for categorical variables. Normality assumptions were assessed using the Shapiro-Wilk test to determine the appropriate statistical approach for the comparisons between groups in relation to the primary and secondary study outcomes. The primary outcome (total OSCE score) was analyzed using an independent samples *t* test after confirming normal distribution. Secondary outcomes (domain-specific OSCE performance) were analyzed using Mann-Whitney *U* tests following confirmation of nonnormal distributions. To control for the increased risk of type 1 error when conducting multiple comparisons across the 5 OSCE domains and ensure that domain-specific findings represent robust effects rather than stochastic observations, Bonferroni correction was applied and yielded an adjusted significance level of a=.01 (0.05/5 comparisons). The Fisher exact test was used to compare pass or fail rates.

Results from independent *t* tests are presented as means and SDs, along with 95% CI for mean differences and Cohen *d* effect sizes. Results from Mann-Whitney *U* tests are presented as means and SDs, and effect size (*r*). Results from Fisher exact tests are presented as frequencies and the corresponding percentage, odds ratio, and 95% CI. The statistical analysis was performed using R (version 4.3.3; R Foundation for Statistical Computing). Differences yielding *P* values <.05 were considered statistically significant, except for secondary outcomes where the Bonferroni-adjusted threshold of *P*<.01 was applied.

### Ethical Considerations

The study was reviewed and approved by the Swedish Ethical Review Authority (reference: 2024-05876-02). Students received both written and verbal information about the study and provided written informed consent before participation. Students could withdraw from the study at any time without providing a reason, and withdrawal had no impact on their educational outcomes. All data were handled in accordance with the General Data Protection Regulation and stored on secure servers at Karolinska Institutet. Audio recordings, transcriptions from feedback generation, and assessments were pseudonymized before analysis. No compensation was provided to participating students.

## Results

### Study Participant Characteristics and Baseline Comparisons

All 115 study participants completed the virtual outpatient clinic and subsequent OSCE-based evaluation, yielding complete data for analysis in all cases. Of the 115 medical students, 61 (53%) were allocated to the AI-generated feedback group and 54 (46.9%) to the control group. Baseline characteristics were well balanced between groups (Table 1). The mean age was similar in the feedback group (mean 26, SD 5.9 years) and the control group (mean 26.8, SD 5.3 years; *P*=.49). Sex distribution yielded no statistically significant difference between the two groups; the feedback group had a numerically greater percentage of female students (35/61, 57.4%) compared with the control group (24/54, 44.4%; *P*=.17). Finally, students’ hospital site distribution was balanced (Table 1).

**Table 1:** Baseline characteristics of the study participants (N=115).

Characteristics		Feedback group	Control group	*P* value	
Age (y), mean (SD)		26.0 (5.9)	26.8 (5.3)	.49	
**Sex, n (%)**					.17
	Female	35 (57.4)	24 (44.4)		
	Male	26 (42.6)	30 (55.6)		
**Hospital site, n (%)**					.85
	KS^a^ Huddinge or DS^b^	31 (50.8)	29 (53.7)		
	KS Solna or SÖS^c^	30 (49.2)	25 (46.3)		

^a^KS: Karolinska University Hospital.

^b^DS: Danderyd Hospital.

^c^SÖS: Södersjukhuset.

### Total OSCE Score Comparisons

**Figure 1:**
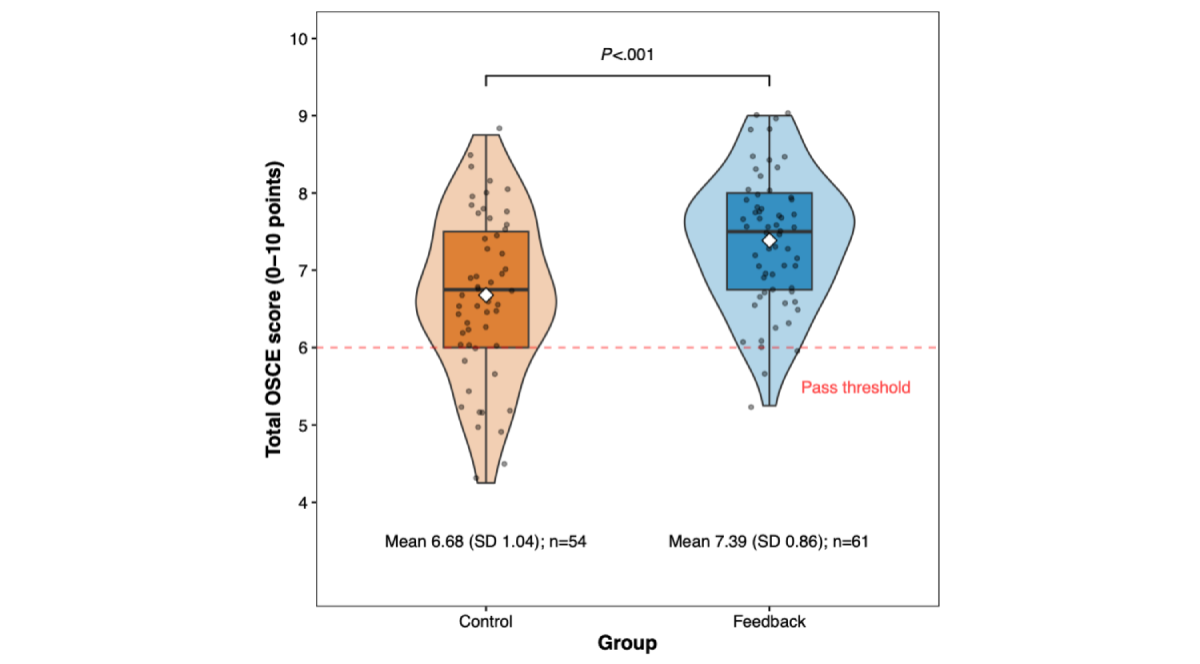
Violin plots illustrating results from the independent samples *t* test comparing the total objective structured clinical examination (OSCE) score between students who received artificial intelligence (AI)–generated feedback following interaction with virtual patients in the Social AI-Enhanced Robotic Interface (SARI) compared with controls. Violin plots display score distributions with embedded boxplots showing the IQR. Individual student scores are shown as jittered points, with means marked by diamonds. The dashed horizontal red line indicates the pass threshold of 6 points.

Students who received AI-generated feedback achieved significantly higher total OSCE scores compared with the control group (mean 7.39, SD 0.86 vs mean 6.68, SD 1.04 points), representing a mean difference of 0.70 points (95% CI 0.35-1.06; t_113_=3.96; *P*<.001). This difference corresponds to a medium to large effect size (Cohen *d*=0.74). Detailed results are presented in Table 2 and Figure 1. No statistically significant differences were noted when comparing results from the 2 assessors (Figure S10 in Multimedia Appendix 1).

**Table 2:** Comparison of objective structured clinical examination performance outcomes between students receiving artificial intelligence–generated feedback following virtual patient interaction with the Social AI-Enhanced Robotic Interface (SARI) and students who did not receive feedback.

Outcomes		Max points	Feedback group (n=61), mean (SD)	Control group (n=54), mean (SD)	Mean difference	Effect size	*P* value
**Primary outcome^a^**							
	Total OSCE^b^ score	10.0	7.39 (0.86)	6.68 (1.04)	0.70	*d*=0.74	*<.001* ^c^
**Secondary outcomes^d^**							
	Communication at start	3.0	2.76 (0.44)	2.62 (0.57)	0.14	*r=*0.14	.13
	Generic medical history	3.5	2.46 (0.65)	2.03 (0.79)	0.43	*r=*0.27	*.004* ^e^
	Targeted medical history	1.5	1.13 (0.22)	1.15 (0.27)	−0.02	*r=*0.05	.60
	Diagnostics and management	1.0	0.59 (0.28)	0.52 (0.25)	0.07	*r=*0.14	.14
	Communication at end	1.0	0.44 (0.46)	0.36 (0.46)	0.08	*r=*0.09	.31

^a^Analyzed using the independent samples *t* test.

^b^OSCE: objective structured clinical examination.

^c^Statistically significant.

^d^Analyzed using Mann-Whitney *U* tests.

^e^After Bonferroni correction (α=.01 for 5 comparisons).

### Individual OSCE Domain Comparisons

**Figure 2:**
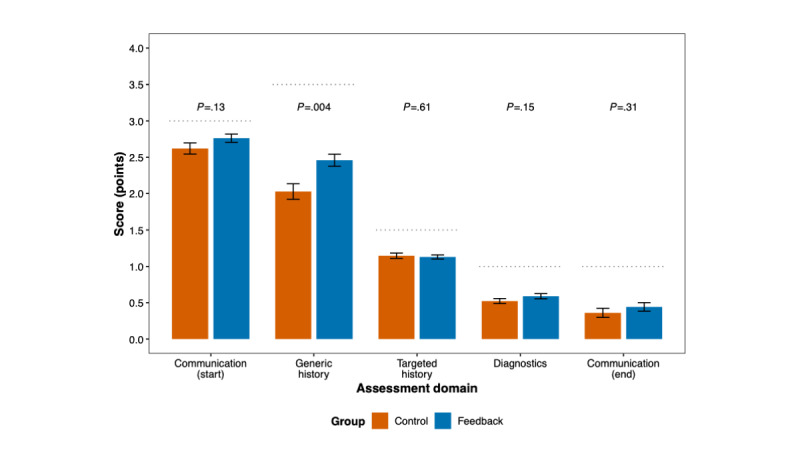
Domain-specific objective structured clinical examination performance across 5 domains of assessment: communication at consultation start, generic medical history, targeted medical history, diagnostics and management reasoning, and communication at consultation end. Bar plots display mean scores and SEs for students receiving artificial intelligence (AI)–generated feedback following interaction with virtual patients in the Social AI-Enhanced Robotic Interface (SARI) vs controls. Dotted horizontal lines indicate maximum scores for each domain. The *P* values from Mann-Whitney *U* tests are displayed above each domain.

Domain-specific analysis using Mann-Whitney *U* tests revealed mixed results across the 5 domains of the OSCE-like assessment. Generic medical history was the only domain that demonstrated a statistically significant difference in favor of the integration of AI-generated feedback; this difference remained significant after applying Bonferroni correction for multiple comparisons (mean 2.46, SD 0.65 vs mean 2.03, SD 0.79 points; *r=*0.27; *P*=.004). No statistically significant differences were observed for communication at consultation start, targeted medical history taking, diagnostics and management reasoning, or communication at consultation end after correction for multiple testing. However, most subsets demonstrated a benefit in favor of the AI-generated feedback group. No statistically significant differences were noted between the assessors. Detailed results are presented in Table 2, Figure 2, and Figure S11 in Multimedia Appendix 1.

### Pass-Rate Comparisons

**Figure 3:**
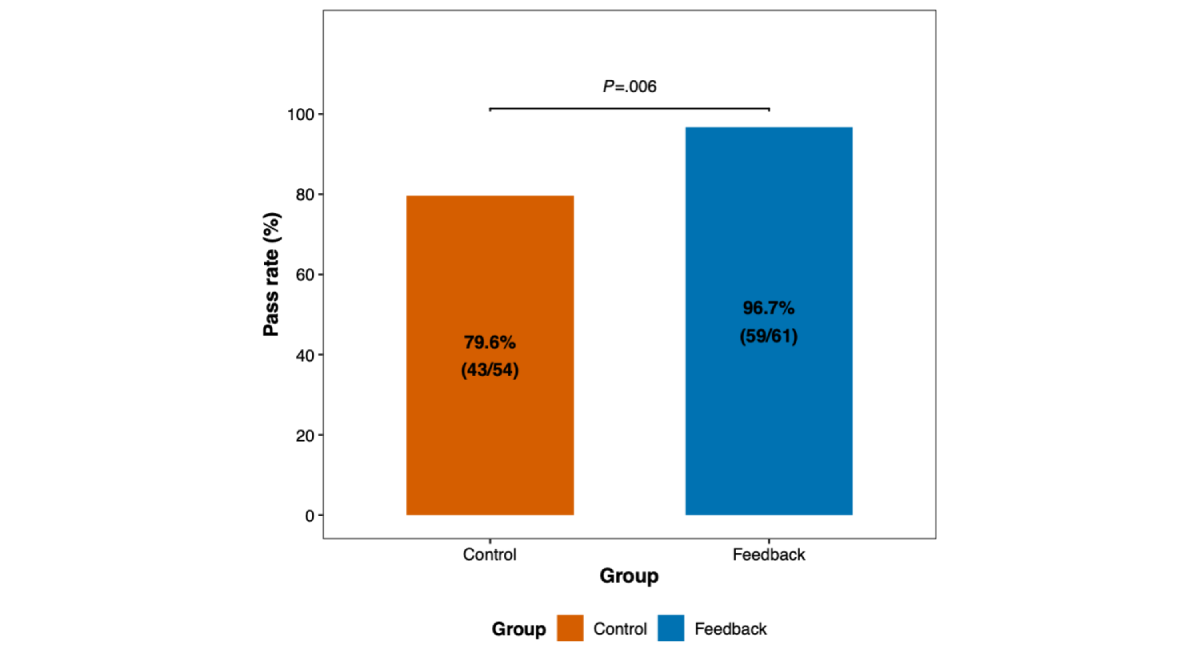
Bar plot illustrating objective structured clinical examination (OSCE) pass rates in students receiving artificial intelligence (AI)–generated feedback following interaction with virtual patients in the Social AI-Enhanced Robotic Interface (SARI) compared with controls. Numbers and percentages are shown within bars. Successful evaluation (pass score) was defined as a total OSCE score of ≥6 points.

Using the predetermined pass threshold of 6 out of 10 points, students who had received AI-generated feedback demonstrated significantly higher pass rates compared with the control group (96.7% vs 79.6%; odds ratio 7.55, 95% CI 1.51-72.2; *P*=.006). This translates to an absolute risk reduction of 17.1 percentage points and a number needed to treat of 6. Detailed results are presented in Figure S12 in Multimedia Appendix 1 and Figure 3. No statistically significant differences in pass rates were noted between the 2 assessors (Figure S13 in Multimedia Appendix 1).

## Discussion

### Principal Findings

This quasi-experimental study provides the first quantitative evidence that the addition of AI-generated feedback on top of expert-led seminars following social robotic VP interactions yields benefits regarding certain aspects of medical students’ performance in subsequent OSCE-based assessments of CR, specifically pertaining to medical history–taking and communication skills. Students receiving AI-generated feedback achieved meaningfully higher total OSCE scores compared with those in the control group, representing a medium to large effect size, while having received feedback was shown to enhance pass rates. The domain-specific improvement in generic medical history suggests that targeted AI feedback effectively enhances systematic medical history–taking competencies, with effects translating from virtual environments to objective clinical assessments.

Our findings build on previous research from our group demonstrating the added value of AI-enhanced social robotic VPs for CR training in medical education [19-22]. Although those earlier studies relied on students’ self-reported perceptions, this study evaluates whether the integration of AI-generated feedback within the SARI translates into measurable improvements in clinical performance compared with training without such feedback. The observed effect size compares favorably with systematic reviews, showing 58% to 72% of VP studies demonstrate positive effects on CR skill training, with particularly strong evidence when VPs include AI-generated feedback that targets specific CR components [6,7].

The factors for improvement likely relate to the immediacy and consistency of AI-generated feedback addressing systematic enquiries during training of medical history taking. Effective feedback principles emphasize the importance of being specific, timely, and actionable [13,14], with research demonstrating that immediate feedback enhances learning retention compared with delayed feedback [15]. As both groups received educational feedback through case-specific follow-up seminars led by consultant rheumatologists, the intervention evaluated the added value of AI-generated written feedback beyond this standard teaching. In addition to nonstructured feedback during these seminars, our AI-system provided additional standardized written feedback immediately following each VP case interaction, likely enhancing retention and skill transfer in the intervention group. While previous studies have shown positive results of integrating LLM-generated feedback in clinical dialogue and VP interaction [16,17], the integration within a social robotic VP platform (SARI) may have created favorable conditions for experiential learning, combining realistic patient interactions with analytical feedback to facilitate skill consolidation through reflective practice [28,33].

Improvements in generic medical history (35% of the total OSCE score), which includes past and current illnesses, lifestyle habits, medications, allergies, and family history, provide important insights into the mechanism of overall performance enhancement. This alignment between the content of the AI-generated feedback, which focused specifically on medical history taking, and the domain where benefit was observed strengthens the specificity of the intervention. Notably, the AI-generated feedback did not address diagnostic accuracy or management reasoning, which may explain why these domains did not show statistically significant differences. However, the sample size may also have limited the statistical power in comparative analyses to yield measurable benefit across domains. This study does not provide direct evidence for improvement in higher-order CR tasks such as differential diagnosis or hypothesis testing. According to a recent systematic review that investigated the role of VPs in providing feedback on CR skills, most feedback systems are designed to target information gathering, while other, more difficult-to-measure aspects of CR largely remain on the research agenda [6].

The domain weighting likely explains why students achieved significantly higher total OSCE scores despite improvements being concentrated to the generic medical history–taking domain. The VP activity design and the virtual outpatient clinic context, which place emphasis on medical history taking, likely create conditions where generic medical history–taking components are particularly responsive to structured feedback. This is in line with previous evidence showing that structured feedback can effectively improve communication skills in medical education [34].

An important consideration is that the AI-generated feedback in our modality is derived from the analysis of transcribed voice interactions between students and the SARI. This suggests that the observed benefits may extend to other voice-enabled VP platforms that facilitate natural conversational interactions. The integration of speech recognition and natural language processing technologies in VP systems may therefore represent an important component for effective AI-generated feedback implementation, warranting further research.

A known risk with LLM use in medical education is the potential for hallucination and factual errors [35]. Our 2-stage feedback algorithm was specifically designed to mitigate this risk. During the first stage, a structured assessment rubric is used to constrain the output of the LLM to predefined evaluation criteria, and during the second stage, feedback is generated based on this structured assessment rather than from unconstrained, noninstructed AI-generated content.

### Limitations and Strengths

The quasi-experimental design with sequential allocation may have introduced selection bias, although baseline characteristics were well balanced between groups. Assessment was limited to 1 OSCE-based rheumatology evaluation, potentially limiting generalizability to other clinical settings and specialties. Although the coauthors portrayed standardized patients, the consultant rheumatologist assessors were blinded to intervention status, minimizing potential bias. Furthermore, there were no statistically significant differences in grading between the assessors, indicating that the standardized OSCE rubric and the clear instructions mitigated the risk of subjectivity for the intended purpose. The single-institution design and use of English for VP interactions and feedback while conducting assessments in Swedish may affect the external validity and represent methodological considerations for future research. Although a statistically significant benefit was only observed in the generic medical history–taking domain, the AI-generated feedback group showed numerical advantages in most assessment domains. This pattern suggests that the study may have been underpowered to detect smaller effect sizes in secondary outcomes. Alternatively, a ceiling effect may have contributed, as both groups generally performed well across other areas of the evaluation rubric.

Subsequent studies should ideally conduct both VP training and assessment in the same language to eliminate potential confounding effects of language switching on clinical performance. This language alignment would provide more precise estimates of AI feedback effectiveness and better reflect real-world clinical education scenarios. Additionally, multicenter studies across different educational contexts and clinical specialties would enhance generalizability, while longitudinal assessment of skill retention and transfer to actual clinical practice would strengthen the evidence.

The study also has several important strengths, including the use of objective clinical performance measures, substantial sample size, and integration with established educational activities. This represents the first study demonstrating that AI-generated feedback in social robotic VPs translates to objectively measured improved medical history taking and CR performance in standardized OSCE-based evaluations.

### Implications

These findings support investment in developing AI feedback systems within medical education curricula, specifically integrated in VP encounters. By systematically improving the quality of medical history–taking training, such systems could contribute to reducing diagnostic errors in clinical practice, where inadequate history taking has been identified as a leading contributor to such errors [10]. However, longitudinal studies following students into clinical practice are required to determine whether the observed benefit in OSCE performance translates to improved patient outcomes. The voice-based nature of student-VP interactions in our study suggests that AI-generated feedback systems may be particularly effective when integrated with conversational VP platforms that enable natural speech communication. This approach could apply across various VP technologies with voice interaction capabilities, potentially expanding beyond social robotic platforms.

### Conclusions

This study demonstrates that AI-generated feedback following social robotic VP interactions yields significant benefit in medical students’ CR performance, particularly in medical history taking, based on standardized OSCE-based assessments. The substantial effect size and greater pass rates among students who received AI-generated feedback provide evidence supporting the integration of AI feedback systems within social robotic platforms for enhancing medical history–taking skills in medical education. The observed differences across domains of the assessment may imply a potential value of domain-specific feedback approaches.

## Appendix

Multimedia Appendix 1 Data on participant recruitment and allocation flowchart, objective structured clinical examination (OSCE)–like assessment rubric, structured history–taking guide with example questions, artificial intelligence (AI)–generated feedback algorithm and prompt configurations for the Social AI-enhanced Robotic Interface (SARI), example AI-generated feedback output, SARI interaction prompt example, translated instructions to students and actors for the OSCE-like assessment, violin plots comparing total and domain-specific OSCE scores between assessors, and tables comparing pass or fail frequencies by feedback condition and by assessor.

## Abbreviations

AI: artificial intelligence
CR: clinical reasoning
LLM: large language model
OSCE: objective structured clinical examination
SARI: Social AI-Enhanced Robotic Interface
VP: virtual patient

## Data Availability

The anonymized datasets generated and analyzed during this study are available from the corresponding author upon reasonable request. Access to data will be granted following appropriate ethical review and data sharing agreements and will require completion of a data transfer agreement and approval from the Swedish Ethical Review Authority, as per Swedish data protection regulations and the European General Data Protection Regulation.
