# Supplementary material for: AI-generated Feedback Following Social Robotic Virtual Patient Interactions and Medical Student Performance: Nonrandomized Quasi-Experimental Study
Source: JMIR Med Educ. 2026 Mar 25;12:e90368. doi: 10.2196/90368 (PMC13062742; doi:10.2196/90368)
Supplement: Multimedia Appendix 1 [file mededu_v12i1e90368_app1.docx]

**AI-generated Feedback in Social Robotic Virtual Patients and Medical Student Performance:**

**Nonrandomized Quasi-Experimental Study**

Alexander Borg, Jonathan Schiött, William Ivegren, Cidem Gentline, Viking Huss,
Anna Hugelius, Benjamin Jobs, Mini Ruiz, Samuel Edelbring,
Carina Georg, Gabriel Skantze, Ioannis Parodis

**Multimedia Appendix 1**

TABLE OF CONTENTS

[Figure S1. Flow chart illustrating recruitment and allocation process. 3](#_Toc222060373)

[Figure S2. The OSCE-like assessment rubric. 4](#_Toc222060374)

[Figure S3. Structured history-taking in rheumatology with example questions. 5](#_Toc222060375)

[Figure S4. Two-stage algorithm system for AI-generated feedback used for the social AI-enhanced robotic interface (SARI). 9](#_Toc222060376)

[Figure S5. Feedback-generating prompt used for the social AI-enhanced robotic interface (SARI). 10](#_Toc222060377)

[Figure S6. Example of the AI-generated feedback provided to students after interaction with the social AI-enhanced robotic interface (SARI). 12](#_Toc222060378)

[Figure S7. Example of prompt used for the social AI-enhanced robotic interface (SARI). 13](#_Toc222060379)

[Figure S8. Instructions to students for the OSCE-like assessment, translated from Swedish. 14](#_Toc222060380)

[Figure S9. Instructions to actors for the OSCE-like assessment, translated from Swedish. 15](#_Toc222060381)

[Figure S10. Violin plots illustrating results from independent samples t-test, comparing total OSCE scoring with assessor 1 (green) compared with assessor 2 (pink). 18](#_Toc222060382)

[Figure S11. Domain-specific OSCE scores across five domains by assessor 1 (green) versus assessor 2 (pink). 19](#_Toc222060383)

[Table S12. Comparisons of pass and fail frequencies between students receiving AI-generated feedback following VP interaction with SARI and students who did not receive feedback. 20](#_Toc222060384)

[Table S13. Comparisons of OSCE pass rate frequencies between assessor 1 (green) versus assessor 2 (pink). 21](#_Toc222060385)

## Figure S1. Flow chart illustrating recruitment and allocation process.

| **Assessed for eligibility (n = 157)** | |  |  |
| --- | --- | --- | --- |
| **↓** | | **→** | **Declined to participate**  **(n = 42)** |
| **Enrolled (n = 115)** | |  |  |
| **↓** | **↓** |  |  |
| Allocated to Intervention:  AI-generated feedback after VP interaction  (n = 61) | Allocated to control:  No AI-generated feedback after VP interaction  (n = 54) |  |  |
| Completed OSCE assessment  (n = 61) | Completed OSCE assessment  (n = 54) |  |  |
| Analysed (n = 61) | Analysed (n = 54) |  |  |

OSCE: objective structured clinical examination ; VP : virtual patient

## Figure S2. The OSCE-like assessment rubric.

| **Communication at the start 3 points** | **Not at all** | **Partially** | **Fully** |
| --- | --- | --- | --- |
| **Begins with open-ended questions and allows the patient space to tell their story**  Does not begin with an open-ended question 0 pts  Begins with an open-ended question but interrupts prematurely 0,5 pts  Begins with an open-ended question and demonstrates appropriate use in relevant situations 1 pts |  |  |  |
| **Clarifies what the patient thinks about their symptoms, whether the patient is worried/concerned about anything, and what the patient hopes to gain from the visit**  Does not clarify 0 pts  Clarifies partially 0,5 pts  Explores thoroughly and provides adequate space for the patient’s thoughts, concerns and expectations 1 pts |  |  |  |
| **Summarises appropriately to ensure information transfer and to validate the patient**  Does not summarise 0 pts  Summarises to some extent 0,5 pts  Summarises at suitable times in a nuanced dialogue with the patient 1 pts |  |  |  |
| **Generic medical history 3,5 points** | **Not at all** | **Partially** | **Fully** |
| **Past/current illnesses**  Not asked 0 pts  Partially asked 0,5 pts  Fully asked 1 pt |  |  |  |
| **Lifestyle habits (smoking, alcohol, physical activity)**  Not asked 0 pts  Partially asked 0,5 pts  Fully asked 1 pt |  |  |  |
| **Current medications and allergies**  Not asked 0 pts  Partially asked 0,5 pts  Fully asked 1 pt |  |  |  |
| **Family history**  Not asked 0 pts  Asked 0,5 pts |  |  |  |
| **Targeted medical history 1,5 points** | **Not at all** | **Partially** | **Fully** |
| **Enquires about back pain (character, location)**  Not asked 0 pts  Partially asked 0,25 pts  Fully asked 0,5 pts |  |  |  |
| **Enquires about extra-articular manifestations (uveitis, enthesitis, gastrointestinal symptoms, etc.)**  Not asked 0 pts  Asks about one type of extra-articular manifestation 0,25 pts  Asks in depth about at least two different forms 0,5 pts |  |  |  |
| **Enquires about morning stiffness**  Not asked 0 pts  Asked but without further exploration 0,25 pts  Asked with follow-up on duration, relieving/aggravating factors 0,5 pts |  |  |  |
| **Diagnostics and management 1 point** | **Not at all** | **Partially** | **Fully** |
| **Diagnostic reasoning**  Faisl to gather information or raise suspicion of joint disease 0 pts  Identifies only peripheral involvement based on history 0,25 pts  Identifies both axial and peripheral involvement based on history 0,5 pts |  |  |  |
| **Further investigations (imaging, blood tests)**  Not mentioned 0 pts  Partially mentioned 0,25p  Provides complete and appropriate investigation proposals 0,5 pts |  |  |  |
| **Communication at the end 1 point** | **Not at all** | **Partially** | **Fully** |
| **Ensures the patient has understood and provides space for additional questions**  Not addressed 0 pts  Addressed to some extent 0,5 pts  Adequately addressed 1 pt |  |  |  |

**Total: / 10 pts**

OSCE: objective structured clinical examination

## Figure S3. Structured history-taking in rheumatology with example questions.

**Reason for consultation**

- What brings you here today?
- What do you hope to get out of the visit today?
- What do you think about your symptoms yourself?
- Do you have any particular concerns or worries about your symptoms?

**Present illness**

**Systemic symptoms**

- Have you experienced fever, low-grade pyrexia, or feverish feelings?
- Have you had any unintentional weight loss? If yes, how much and over what period?
- Have you experienced night sweats?
- Do you feel generally unwell/do you have a general feeling of malaise?
- Have you had any clots (thromboembolic events)?

**Musculoskeletal symptoms**

- Do you have muscle pain?
- How would you describe the pain?
- Where is the pain located?
- Does the pain radiate anywhere?
- Is the pain symmetrical?
- Do you have claudication (pain on exertion) in your arms?
- Do you have joint pain?
- Where is the joint pain located (number, localisation)?
- Is it symmetrical?
- Do you have swollen joints?
- Do you have any sausage-like swelling in fingers or toes (dactylitis)?
- Do you have pain where muscles/tendons attach (enthesitis)?
- Do you have back pain?
- When did it start? At what age?
- Does it have inflammatory characteristics?
- How does the pain vary throughout the day?
- How is it affected by rest?
- Does it improve with movement or exercise?
- Do you experience morning stiffness?
- How long does it last?
- Do you have muscle weakness?
- Is it proximal or distal?
- How do your symptoms vary throughout the day?
- Do you have difficulty performing daily activities or do you need support?

**Other organ systems**

**Head/face**

- Do you have any new-onset headache?
- Have you experienced any new and severe hair loss?
- Do you have tenderness over the temporal artery?
- Do you have jaw claudication (pain when chewing)?
- Do you have recurrent sinusitis?
- Do you have nosebleeds or nasal crusts?
- Have you experienced ulcers or blisters in your mouth?
- Do you have reduced hearing?
- Do you have difficulty swallowing?
- Have you experienced symptoms from the nervous system (e.g., CNS: headache, seizures, et cetera)?

**Eyes**

- Have you experienced visual changes?
- Do you have visual loss?
- Do you have double vision?
- Do you have blurred vision?
- Do you have eye problems such as red eye or light sensitivity?
- Have you experienced dryness in eyes and/or mouth?

**Respiratory system**

- Do you have lung symptoms?
- Do you have a cough?
- Have you coughed up blood (haemoptysis)?
- Do you have shortness of breath?

**Skin**

- Do you have any skin rashes?
- Have you been examined by a dermatologist?
- Do you have psoriasis?
- Do you have specific rashes (Gottron’s sign, shawl sign)?
- Do you have Raynaud’s phenomenon?
- Mono-, bi- or triphasic?
- For how long?
- Do you experience photosensitivity?

**Abdomen/urinary tract**

- Do you have any gastrointestinal symptoms?
- Do you have any kidney or urinary tract problems?
- Have you noticed any changes in bowel or urinary habits?

**Reproductive organs**

- Ulcers/blisters in the genital area?
- Miscarriages?
- How many?
- Early or late in pregnancy?

**Past medical history**

- Do you have any previously treated conditions?
- Have you undergone any operations?

**Family history**

- Do you have any family history of:
  - Rheumatic diseases?
  - Muscle diseases?
  - Psoriasis?
  - Spondylitis?
  - Cancer?

**Lifestyle**

- Do you smoke? Quantify
- Do you drink alcohol? Quantify
- How physically active are you? Quantify
- What are your eating habits like?
- How do you sleep?

**Social circumstances**

- Who are your closest relatives?
- How do you live?
- What do you work as?

**Current medications**

- What medications are you currently taking?
- Have you taken NSAIDs or other analgesics?
- How effective are your medications?

**Allergies**

- Do you have any allergies?

##
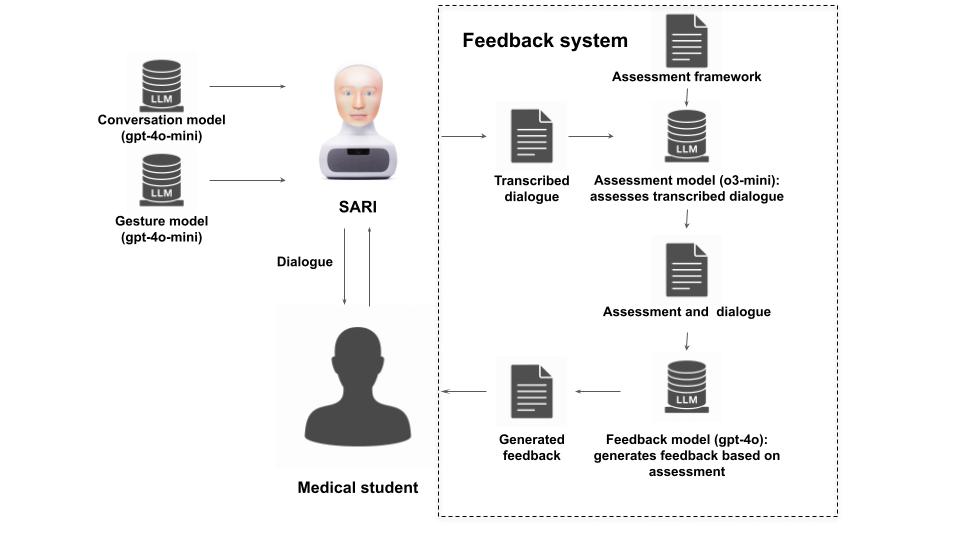
Figure S4. Two-stage algorithm system for AI-generated feedback used for the social AI-enhanced robotic interface (SARI).

AI: artificial intelligence; LLM: large language model; SARI: social AI-enhanced robotic interface.

## Figure S5. Feedback-generating prompt used for the social AI-enhanced robotic interface (SARI).

Act as a medical professor and an expert in giving feedback to students, that should give personal feedback to a medical student on the following dialogue. Address the student with **“**you**”**. Use simple English, except if you need more complex language for medical terms. Also, be careful in using formulations that include adjectives such as **“**important**”**, **“**essential**”**, etc. when addressing different objectives. It is better to do it more objectively such as **“**You did not do X, it could have been done to explore Y because of Z**”**.

The situation is the following. A medical student acts as a doctor and receives a patient with a rheumatic condition. It is up to the student to ask questions to the patient and learn about the patient**’**s situation to be able to diagnose the patient.

Your aim is to give feedback in a structured manner on the student**’**s clinical reasoning and ability on asking questions to learn about the patient’s situation. Your task is to coach them to be more prepared when encountering a similar situation. However, it is important that you do not give away or mention what condition the patient have.

To your help you have the full dialogue between the medical student and the patient, as well as a grading file that assesses whether the student has asked all the important questions about the patient’s situation or not. The questions and the grading are the knowledge requirements. You also have a definition on what clinical reasoning is in this context. Focus both on the not fulfilled questions, i.e., the graded answer is **“**no**”**, **“**partially**”** or **“**N/A**”** to give constructive feedback on what the student can improve, and the fulfilled questions, i.e., the graded answer is **“**yes**”** to give positive feedback on what the student did well. Important is that that you do not cover the questions one by one. Instead give more general bullet points merging the missed questions and the fulfilled questions, without stating the questions in the feedback.

The first five questions you can merge into one bullet point and give general feedback on, both constructive and positive (i.e., 1. Did the student ask open-ended questions initially? 2. Did the student ask about the patient's own thoughts regarding their symptoms?, 3. Did the student ask the patient about specific concerns/worries about their symptoms?, 4. Did the student ask what the patient hopes to gain from the visit?, 5. Did the student ask if the patient has any past or current disease**/**worries?). These questions are not the most relevant in diagnosing the patient and a merging of them leaves more room for the more rheumatic focused questions coming in question six and forward. Treat this bullet point as one of the others, i.e., do not mention anything about the questions.

Use quotes from the Dialogue as often as possible to contextualize the feedback. Please provide a detailed explanation of your reasoning for the feedback in a step-by-step, chain-of-thought format.

**Definition on Clinical Reasoning:**

When grading the students clinical reasoning, assess both analytical and non-analytical (pattern recognition) thinking processes if possible. Consider both diagnostic reasoning **(**the ability to come closer towards diagnostically accurate responses) and management reasoning (contextual decision-making with multiple valid approaches). These processes sometimes overlap. Define your assessment criteria clearly and be systematic, remembering that there isn**’**t always a single **“**correct**”** answer. Grade students on their ability to integrate knowledge with practical decision-making.

**Dialogue** (Use quotes from this dialogue as often as possible to contextualize the feedback):

{str(dialogue_text)}

**Grading:**

{str(grading_file)}

**Response format:**

Bullet Point Header:

A detailed feedback text that focuses on both the not fulfilled questions (constructive feedback) and the fulfilled questions (positive feedback) and does not mention what condition the patient has. For each bullet, explain why you give this feedback. Use quotes from the Dialogue as often as possible to contextualize the feedback.

## Figure S6. Example of the AI-generated feedback provided to students after interaction with the social AI-enhanced robotic interface (SARI).


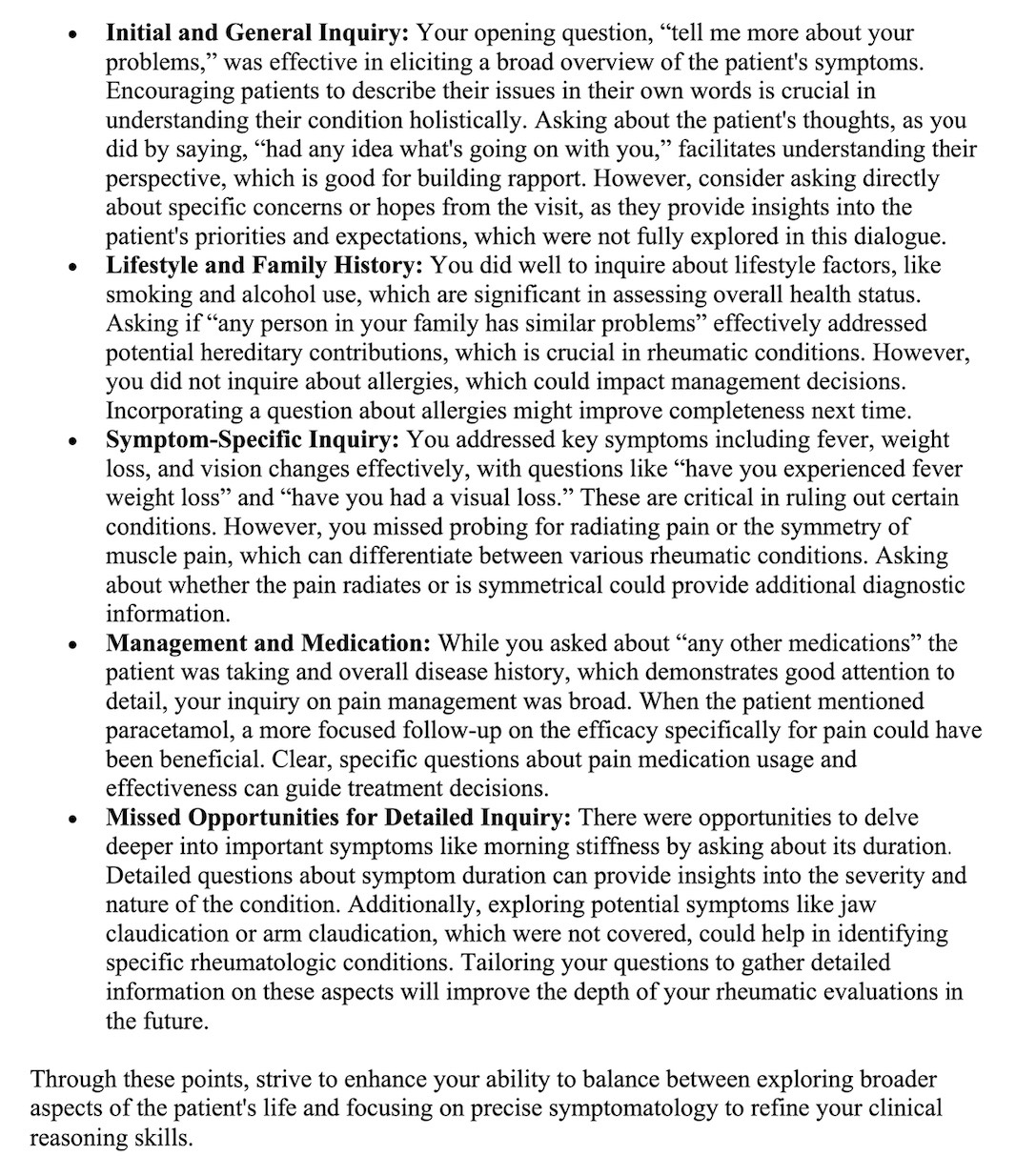


## Figure S7. Example of prompt used for the social AI-enhanced robotic interface (SARI).

Mikael is about to meet his physician for the first time. Mikael is a 68 years old man who made an appointment a few days back. Apart from the age and sex of the patient, the only information the physician has is that Mikael has sought care because of “ache in the body”.

**The following is some information about Mikael’s condition [shortened]:**

- He has always been healthy and has not felt any pain similar to that he is seeking for now.
- Sometimes, he experiences back pain, but it has never been present for that long and it has not had the same character.
- In Mikael’s medical charts, it is stated that his blood lipids are above normal.
- Mikael has been advised to eat healthier to avoid developing diabetes and lower his blood lipids.
- Mikael has had a stable blood pressure since he has been on anti-hypertensive treatment.

**The following is a dialogue between Mikael and his physician:**

- Mikael: Hi doctor.
- Physician: Hello Mikael, my name is Morgan.
- Mikael: Nice to meet you, Morgan. Thanks for seeing me.
- Physician: Of course! Could you describe why you are here today?

**Write the next line that Mikael would say.**

## Figure S8. Instructions to students for the OSCE-like assessment, translated from Swedish.

**OSCE station: Rheumatological history**

**Scenario**

You are working as a junior doctor in a rheumatology outpatient clinic. Your next patient is Christian Andersson, 32 years old, who is attending for a first consultation due to back pain. Because of time constraints, you have only 8 minutes to take a focused history and provide preliminary advice to this worried patient.

Your task

1. Take a structured but focused history regarding relevant rheumatological aspects
2. Assess the patient’s concerns and expectations
3. Suggest resasonable initial investigations and management

*Good luck!*

OSCE: objective structured clinical examination

## Figure S9. Instructions to actors for the OSCE-like assessment, translated from Swedish.

**Patient instructions: Christian with axial spondylarthritis**

**The patient**

- Christian Andersson, 32 years old
- IT developer at a start-up in Stockholm
- Lives with partner Sara, 4 years together
- Previously very active with running/gym

**Standard response to opening question (why are you here?)**

“I’ve had problems with my back for about two years now, and it’s been getting worse. It’s particularly bad in the mornings”

**Main symptoms (to volunteer spontaneously)**

- Back pain that has been ongoing for some time but is now becoming unbearable
- Difficulty sitting for long periods, often works from home
- No longer able to run as before

**Only mention if specifically asked**

*Back symptoms*

- Location: Lower back extending up towards the thoracic spine
- Character: Dull, aching pain
- Stiffness worst in the lower back
- Mornin stiffness requiring 1–2 hours to loosen up
- Night pain waking you around 3–4 a.m.
- Relieving factors: Improves with movement and a hot shower
- Daily variation: Worst in the morning but improves during the day and with activity

*Other joints/areas*

- Right knee: swollen for the past 6 months
- Left Achilles tendon: painful
- Right big toe: swollen and tender

*Other symptoms*

- Eyes: two episodes of redness/irritation in the past year
- Bowel: intermittent loose stools
- Weight: lost 3 kg over 6 months unintentionally

*Past medical history*

- Appendectomy as a teenager
- A couple of ankle sprains related to running
- Otherwise healthy

*Family history*

- Father: “some form of rheumatism”
- If specifically asked about psoriasis: aunt has it

*Lifestyle*

- Previously: training 4–5 times/week
- Now: mostly walking
- Non-smoker
- Alcohol: 1–2 glasses of wine/weekend

*Medications*

- Ibuprofen when needed for pain relief (helps but effect wears off)
- No other medications/allergies

**How you should act**

*If asked about concerns/thoughts*

- Show concern about ability to work
- Ask about treatment options
- Do NOT mention concern about cancer

*If the student summarises*

- Confirm what is correct
- Correct mistakes politely
- Show uncertainty if no summary is given

*If the student suggests actions*

- Show interest
- Ask about blood tests/scans
- Ask about prognosis/exercise advice

**Important to remember**

1. Give short answers to closed questions
2. Provide more detail with open questions
3. ALWAYS wait for specific questions about:
   - Problems outside the back
   - Eye problems
   - Family history
   - Bowel problems

## Figure S10. Violin plots illustrating results from independent samples t-test, comparing total OSCE scoring with assessor 1 (green) compared with assessor 2 (pink).

OSCE: objective structured clinical examination.

## Figure S11. Domain-specific OSCE scores across five domains by assessor 1 (green) versus assessor 2 (pink).

 Results derive from Mann-Whitney *U* tests.

OSCE: objective structured clinical examination.

## Table S12. Comparisons of pass and fail frequencies between students receiving AI-generated feedback following VP interaction with SARI and students who did not receive feedback.

| **Group** | **Total N** | **Passed, n (%)** | **Failed, n (%)** | **Difference** | **OR (95% CI)** | ***P* value** |
| --- | --- | --- | --- | --- | --- | --- |
| **Feedback group** | 61 | 59 (96.7%) | 2 (3.3%) |  |  |  |
| **Control group** | 54 | 43 (79.6%) | 11 (20.4%) |  |  |  |
| Difference |  |  |  | 17.1 PP | **7.55 (1.51–72.2)** | **.006** |

Data are presented as numbers and percentages. Statistical analysis was performed using Fisher’s exact test. Number needed to treat = 6 students. Statistically significant *p* values are in bold.

CI: confidence interval; OR: odds ratio; PP: percentage points. SARI: Social AI-enhanced Robotic Interface

## Figure S13. Comparisons of OSCE pass rate frequencies between assessor 1 (green) versus assessor 2 (pink).

Results derive from Fisher’s exact test.

OSCE: objective structured clinical examination.
